# Hepatorenal syndrome misdiagnosis may be reduced using inferior vena cava ultrasound to assess intravascular volume and guide management

**DOI:** 10.1080/0886022X.2023.2185468

**Published:** 2023-03-03

**Authors:** Elaine M. Kaptein, Zayar Oo, Matthew J. Kaptein

**Affiliations:** a Departments of Medicine, Divisions of Nephrology, University of Southern California, Los Angeles, CA, USA; b Loma Linda University Medical Center, Loma Linda, CA, USA

**Keywords:** Inferior vena cava ultrasound, intravascular volume, acute kidney injury, hepatorenal syndrome, volume management

## Abstract

Hepatorenal syndrome (HRS) is a diagnosis of exclusion defined as acute kidney injury (AKI) with cirrhosis and ascites, with serum creatinine unresponsive to standardized volume administration and diuretic withdrawal. Persistent intravascular hypovolemia or hypervolemia may contribute to AKI and be revealed by inferior vena cava ultrasound (IVC US), which may guide additional volume management. Twenty hospitalized adult patients meeting HRS-AKI criteria had IVC US to assess intravascular volume after receiving standardized albumin administration and diuretic withdrawal. Six had IVC collapsibility index (IVC-CI) ≥50% and IVCmax ≤0.7 cm suggesting intravascular hypovolemia, 9 had IVC-CI <20% and IVCmax >0.7 cm suggesting intravascular hypervolemia, and 5 had IVC-CI ≥20% to <50% and IVCmax >0.7 cm. Additional volume management was prescribed in the 15 patients with either hypovolemia or hypervolemia. After 4–5 days, serum creatinine levels decreased *≥*20% without hemodialysis in 6 of 20 patients – 3 with hypovolemia received additional volume, and 2 with hypervolemia plus one with ‘euvolemia’ and dyspnea were volume restricted and received diuretics. In the other 14 patients, serum creatinine failed to persistently decrease ≥20% or hemodialysis was required indicating that AKI did not improve. In summary, fifteen of 20 patients (75%) were presumed to have intravascular hypovolemia or hypervolemia by IVC ultrasound. Six of the 20 patients (40%) improved AKI by 4-5 days of follow-up with additional IVC US-guided volume management, and thus had been misdiagnosed as HRS-AKI. IVC US may more accurately define HRS-AKI as being neither hypovolemic nor hypervolemic, and guide volume management, decreasing the frequency of HRS-AKI misdiagnosis.

## Introduction

Acute kidney injury (AKI) is common in patients with cirrhosis and ascites, with a prevalence in large studies ranging between 27% and 53%, and is attributed to acute tubular necrosis (ATN) in 14–35%, to hypovolemia in 27–50%, and to AKI secondary to hepatorenal syndrome (HRS-AKI) in 15–43% of all cases [1,2]. HRS-AKI is characterized by constriction of the renal arteries, resulting in decreased kidney blood flow, in the absence of substantial abnormalities in kidney histology [1]. By definition, HRS-AKI, also termed HRS-1 [1], is a diagnosis of exclusion of other causes of AKI including other pre-renal, intra-renal and post-renal disorders [1].

A recent systematic review and meta-analysis by Ning et al. [3], indicates that the presence of AKI in patients with cirrhosis is significantly associated with higher mortality in-hospital (OR 5.92), at 30 days (OR 4.78), and at 1 year follow-up (OR 4.82) compared to patients without AKI. In these patients, increasing risk of mortality is correlated with an increase in the stage of AKI. These findings suggest that early detection and prompt management of potentially reversible causes of AKI, such as persistent intravascular hypovolemia or hypervolemia after standardized volume administration and diuretic withdrawal, may decrease the severity of AKI and be associated with improved clinical outcomes.

Hospitalized patients with cirrhosis, ascites, and AKI are frequently not in steady-state and may have mismatch between decreased intravascular volume – due to increased capillary permeability, lactulose-induced diarrhea, gastrointestinal hemorrhage, therapeutic paracentesis, decreased oral sodium intake and/or administration of diuretics – in the face of increased extravascular volume, as evidenced by edema and ascites due to cirrhotic pathophysiology. HRS-AKI has been reported to be more likely when AKI fails to improve after at least two days of intravenous albumin of 1 g/kg of body weight per day to a maximum of 100 g/day together with diuretic withdrawal [1]. This regimen is prescribed for patients suspected to have hypovolemic AKI with cirrhosis and ascites in an attempt to restore intravascular euvolemia and renal perfusion. This standardized volume repletion may be inadequate to completely correct intravascular volume depletion or may cause intravascular volume overload, which in turn may induce AKI unrelated to HRS. Nearly one third of patients with cirrhosis receiving standardized albumin infusions develop volume overload [4]. Intravascular volume excess may exacerbate latent or overt cirrhotic cardiomyopathy [4,5] or intra-abdominal hypertension (IAH) causing AKI not due to HRS [6], which may respond to volume removal.

By definition, HRS-AKI is not responsive to standardized volume expansion and discontinuation of diuretics [1,2], after which intravascular volume repletion is presumed to have been achieved. However, assessment of intravascular volume determined from the clinical history, physical examination, and laboratory and radiological testing has low sensitivity and/or specificity and may not accurately detect intravascular volume depletion or excess [7,8], particularly in hospitalized patients with ascites and edema due to cirrhosis. Inferior vena cava ultrasound (IVC US) may be useful to assess intravascular volume following standardized volume administration and withdrawal of diuretic therapy in patients assumed to have HRS-AKI, and these patients may benefit from further volume management to improve AKI. In one study [9], 64% of the patients assumed to have HRS-AKI were subsequently found to have intravascular hypovolemia or hypervolemia, or to have IAH, by IVC US assessment, and 35% of these had improvement in AKI in the subsequent three to five days with IVC US-guided volume management, thus making a diagnosis of HRS-AKI unlikely.

We postulate that some of our patients with cirrhosis and AKI, assumed to have HRS-AKI because they did not improve renal function after standardized volume administration and diuretic withdrawal, may instead have had persistent intravascular volume depletion or may have developed intravascular volume overload as assessed by IVC US, contributing to AKI. AKI may improve with additional IVC US-guided volume management making the diagnosis of HRS-AKI unlikely.

## Methods

### Patient population and selection criteria

Approval for this study was obtained from the University of Southern California Institutional Review Board (HS-12-00383). Procedures were followed in accordance with the ethical standards of the University of Southern California Institutional Review Board and with the Declaration of Helsinki of 1975, as revised in 2000. Written informed consent was not required for the ultrasound procedure since this was performed for clinical purposes, as was subsequent volume management, and data collection was retrospective.

**Figure 1.**
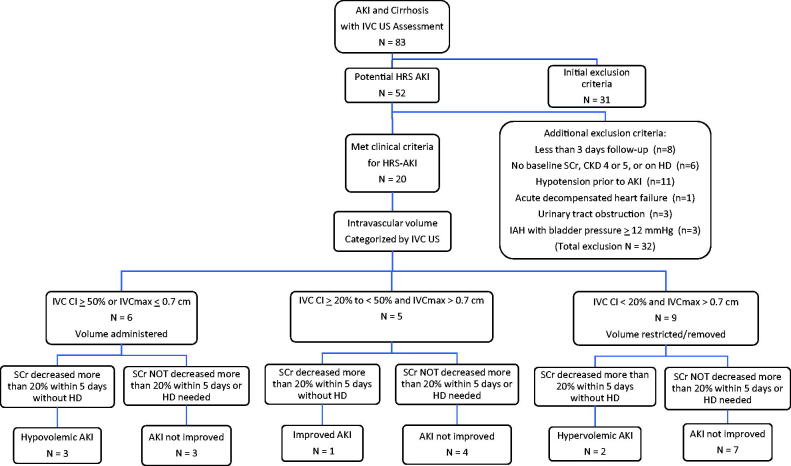
Summary of patients with possible HRS-AKI at the time of the nephrology consult and subsequent follow-up. The selection of patients who met clinical criteria for HRS-AKI was based on detailed chart review including having received standardized volume administration while withholding diuretics. Intravascular volume was defined by the initial IVC US at the time of consult. Changes in serum creatinine levels with subsequent volume management were assessed during the first 5 days of follow-up after the initial IVC US. Definition of response to therapy is a ≥20% decrease in serum creatinine from the peak value [9]. Abbreviations: AKI: acute kidney injury; CKD: chronic kidney disease; CI: collapsibility index; HD: hemodialysis; HRS: hepatorenal syndrome; IAH: intra-abdominal hypertension; IVC: inferior vena cava; SCr: serum creatinine concentration; US: ultrasound.

Adult patients hospitalized between 1 August 2012 and 28 February 2020, with AKI, cirrhosis and ascites, who had an IVC US performed, were evaluated for HRS-AKI defined by current criteria [1]. Eighty-three patients were retrieved from the daily renal consult rounding lists of the primary investigator (Figure 1). Initial exclusion criteria adapted from the Practice Guidelines by the American Association for the Study of Liver Diseases [1] included documentation of potential causes of AKI other than HRS-AKI such as: (1) recent cardiopulmonary arrest, hypotension with vasopressor support, (2) current or recent use of nephrotoxic agents, (3) evidence of glomerulonephritis or interstitial nephritis, and/or (4) urinary tract obstruction. Other exclusion criteria included: (5) IVC thrombosis, (6) CKD stage 4 or 5 or maintenance dialysis therapy prior to or at the time of IVC US, (7) preexisting acute decompensated heart failure or severe cardiac valvular disease, which may alter IVC US findings [10] and also impair renal function, and/or (8) patients who had not received the standardized albumin administration and diuretic withdrawal prior to the IVC US. Thirty one patients were excluded based on these criteria.

The remaining 52 patients without exclusion criteria clearly documented on the rounding lists were further evaluated by detailed chart review for the presence of any of the above listed plus additional exclusion criteria (Figure 1). Thirty-two more patients were excluded due to these exclusion criteria: (1) follow-up for <3 days after the IVC US since they may not have had time to receive or respond to the volume management recommendations, (2) had recently received hemodialysis therapy, (3) did not have a documented baseline serum creatinine value, (4) had urinary retention documented by bladder catheterization, and/or (5) had tense ascites and IAH with bladder pressures >12 mmHg [6].

The remaining 20 patients also had to have a persistent increase in serum creatinine of ≥ 0.3 mg/dL within 2 days of onset of AKI or alternately an increase in serum creatinine of ≥50% from a baseline value documented within the prior 90 days, and failure of serum creatinine to persistently decrease ≥0.3 mg/dL after at least 2 days of diuretic withdrawal and a trial of volume expansion with albumin (1 g/kg of body weight per day to a maximum of 100 g/day) [1]. These 20 patients, who met the above criteria for HRS-AKI (Figure 1) and had received volume expansion and diuretic withdrawal prior to the IVC US examination, had been presumed to be intravascularly replete by the primary team.

### Bedside ultrasonography

At the time of the nephrology consultation, all patients had bedside US evaluation in a semi-recumbent position at an incline of 30–45 degrees without Valsalva or sniff maneuvers using a portable US machine (LOGIQ e B12, GE Healthcare, Wauwatosa, WI 53226) as previously described [10]. The heart was viewed to assess contractility and whether a pericardial effusion was present. The IVC was imaged *via* the subcostal window in a longitudinal plane using a 3.5-MHz curvilinear probe, using B mode, performed or directly supervised by the primary author. IVC US data were only included if the IVC was adequately visualized to allow measurements of the respiratory variations of the IVC diameter. IVC collapsibility index (CI) was calculated as

(IVCmax–IVCmin)/IVCmax×100%.

### Patient classification, volume management recommendations, and follow-up

Volume management recommendations for the 20 selected patients were based on the clinical, laboratory and radiological findings, taking into consideration the IVC US data (Figure 1). Patients with very small IVCmax values (≤0.7 cm) may have small changes in IVC diameters with respiration and still may be hypovolemic [11]. If the IVC CI was ≥50% or the IVCmax was ≤0.7 cm, suggesting hypovolemia [10,11], additional volume administration was recommended as clinically indicated and documented as received. If the IVC CI was <20% and the IVCmax was >0.7 cm, suggesting intravascular volume overload [10,11], volume as saline, albumin and/or blood products was restricted, and diuretics, therapeutic paracentesis, and/or ultrafiltration were recommended and received as clinically indicated. Those with IVC CI ≥20–<50% and IVCmax >0.7 cm were assumed to be neither overtly hypovolemic nor hypervolemic and had ongoing volume losses estimated and replaced taking into account the isonatremic equivalents of body fluid losses and parenteral and enteral inputs [12], with clinical, laboratory and radiological findings taken into consideration.

Available serum creatinine levels from the earliest recorded value until the final follow-up time were obtained from medical records. The change in serum creatinine from peak values within 3 days before or after the IVC US until 4–5 days after the IVC US, in the absence of hemodialysis therapy, was used to assess response to post-IVC US volume management. *A* ≥ 20% decrease in serum creatinine from the peak value within 48–72 h of the therapeutic intervention [9], not resulting from hemodialysis therapy, was defined as hypovolemic- or hypervolemic-AKI [1]. An increasing serum creatinine or a decrease <20% from the peak value, or requirement for dialysis therapy, was defined as AKI not responsive to volume management [1,9].

## Results

As shown in Figure 1, only 20 hospitalized adult patients with cirrhosis and ascites, without other identifiable causes of AKI, met all criteria for a clinical diagnosis of HRS-AKI after receiving standardized volume administration with albumin and withdrawal of diuretics, followed by an IVC US evaluation. Thirteen patients were in the intensive care unit and seven were on the medical wards.

Tables 1 and 2 show the clinical characteristics for the 20 patients who met all clinical criteria for HRS-AKI. As shown in Figure 1 and Table 2, six patients with IVC CI ≥50% or IVCmax ≤0.7 cm, consistent with intravascular hypovolemia, received additional volume within the 4–5 days of follow-up (Table 3). Four of five patients with IVC CI ≥20–<50% and IVCmax > 0.7 cm, inconsistent with intravascular hypovolemia or hypervolemia, received volume replacement during follow-up based on estimated isonatremic total body volume losses and parenteral and enteral inputs [12,13]. Patient #7 with IVC CI of 24% and IVCmax >0.7 cm had shortness of breath and was therefore volume restricted and given diuretics. Nine patients had IVC CI <20% and IVCmax > 0.7 cm, consistent with intravascular volume overload, and had volume restriction, intravenous diuretics, therapeutic paracentesis and/or ultrafiltration recommended and carried out at the discretion of the primary team (Table 3).

**Table 1.** Characteristics of patients who met clinical criteria for HRS-AKI (1) and then received IVC US guided volume management and follow-up for at least 5 days.

	Total meeting clinical criteria for HRS-AKI	AKI responsive to volume management^e^	Unable to exclude HRS-AKI (No improvement of AKI by 5th day of follow-up)^e^
Number	20	6	14
Age [years] [median, range]	56.5 (28–73)	50 (28–63)	60 (30–73)
Gender [M]	15	5	10
Baseline SCr (mg/dL) [median, range]^a^	0.96 (0.52–2.57)	1.33 (0.85–2.14)	0.95 (0.52–2.57)
At time of initial consult and IVC US			
Peak SCr (mg/dL) [median, range]^b^	3.85 (1.60–17.20)	3.07 (1.60–4.11)	5.07 (1.61–17.20)
MELD score [median, range]	34 (22–43)	35 (28–40)	34 (22–43)
Cause of cirrhosis/ESLD^c^			
Ethanol	16	6	10
Hepatitis B and/or C	3	1	2
Cryptogenic	4	0	4
Subsequent course			
Total follow-up days^d^ [median, range]	14 (3–57)	14 (5–54)	15.5 (3–57)
Hospital survival [Yes, %]	12 (60%)	3 (50%)	9 (64%)

Abbreviations: AKI: acute kidney injury; HRS: hepatorenal syndrome; IVC US: inferior vena cava ultrasound; MELD: model for end-stage liver disease (ESLD) (per OPTN policy, January 2016, pp. 4–5); SCr: serum creatinine.

^a^Baseline serum creatinine is the lowest value available within the previous 3 months prior to consult.

^b^Peak serum creatinine value immediately prior to or within 3 days of the consult and initial IVC US.

^c^Patients may have had more than one cause of ESLD as per Table 2.

^d^Days followed after initial consult and IVC US.

**^e^**Improvement of AKI is defined as decrease of serum creatinine ≥20% without hemodialysis therapy, and no improvement defined as failure of serum creatinine to decrease ≥20% or requirement for dialysis therapy (9), after IVC ultrasound guided volume management.

**Table 2.** Individual data for patients with clinical criteria for HRS-AKI after standardized albumin administration plus diuretic withdrawal followed by IVC US guided volume management.

					Prior to initial IVC US		Within ±3 days of initial IVC US^c^		Follow-up 4 to 5 days after IVC US						
Pt ID	Age years	Sex M/F	IVC Max^a^ (cm)	IVC CI^a^ (%)	Baseline SCr^b^ (mg/dL)	Day of baseline SCr	Peak SCr^c^ (mg/dL)	Day of peak SCr	Lowest SCr^e^ (mg/dL)	Day of lowest SCr	Reason for admission	Secondary Disorders	MELD score	Hospital survival (Y/N)	Total days of follow-up
HYPOVOLEMIA (IVC CI ≥50% or IVCmax ≤0.7 cm) (*n* = 6)															
Hypovolemic AKI (*n* = 3)															
1	43	M	1.25	100^g^	1.97	−14.0	4.11	−1.8	1.72	4.7	Acute GI bleed	1,3a,11	39	Y	25
2	49	M	1.42	58	0.85	−48.1	1.60	+0.9	1.13	3.1	Abdominal pain	1,3a,10	30	Y	54
3	55	M	0.50	100^g^	2.14	−2.9	3.46	−0.8	2.49	4.8	AMS	1,3a,10,11	40	N	14
Unable to exclude HRS-AKI^d^ (n = 3)															
4	73	M	1.00	50	0.77	−14.2	6.27	+0.8	N/A	N/A	AMS	1, 3a, 9,10	43	Y	18
5	64	M	0.50	100^g^	0.90	−22.5	2.15	+2.8	2.10	4.8	AMS, large volume paracentesis	2,3a,3b,4,7,11	32	Y	22
6	45	M	0.68	63	0.88	−1.3	4.02	+2.9	5.58	4.9	AMS	1,3a,10	39	N	7
NOT HYPOVOLEMIA nor HYPERVOLEMIA (IVC CI ≥20% to <50% and IVCmax >0.7 cm) (n = 5)															
Possible hypervolemic AKI (*n* = 1)															
7	28	M	2.50	24	0.86	−1.5	2.97	+1.6	2.13	4.9	Acute decompensated liver failure	1,3a,7,10,11	40	Y	12
Unable to exclude HRS-AKI^d^ (*n* = 4)															
8	55	M	2.10	40	2.24	−2.1	3.58	+1.9	N/A	N/A	Acute decompensated liver failure	1,2,3c	22	N	13
9	63	M	1.19	27	0.53	−76.1	14.38	+2.8	N/A	N/A	Acute GI bleed	1,3a,4,5,7,10	33	Y	57
10	58	M	1.76	36	0.99	−4.3	2.63	+1.3	N/A	N/A	Abdominal pain/distension	1,3a,3b,4,5,7,10,11	40	Y	36
11	30	F	2.00	27	1.38	−4.8	2.79	+2.7	N/A	N/A	AMS	2,3a,10,12	39	N	6
HYPERVOLEMIA (IVC CI <20% and IVCmax >0.7) cm (*n* = 9)															
Hypervolemic AKI (*n* = 2)															
12	51	M	3.45	3	0.93	−7.5	4.59	+1.5	3.35	5.0	Acute GI bleed	1,3a,4,7,11	28	N	6
13	63	F	2.97	3	1.72	−3.9	3.03	+1.2	2.32	4.9	Acute EtOH withdrawal	1,3a,3b,7,8,10,11	31	N	14
Unable to exclude HRS-AKI^d^ (n = 7)															
14^f^	70	F	1.88	14	0.77	−54.9	1.61	−2.1	1.55	4.0	GI bleed, anemia	2,3a,10,12	32	N	3
15^f^	62	M	1.92	1	2.57	−12.2	6.73	−1.3	N/A	N/A	Abdominal pain, AMS	1,3c,10	34	Y	26
16	63	F	1.87	17	0.64	−53.8	2.71	+1.7	3.30	4.7	Acute GI bleed	2,3a,4,5,6,10	36	Y	12
17	40	M	1.78	10	0.52	−34.5	6.90	+1.2	N/A	N/A	SBP	1,3a,7,8,10	40	N	8
18^f^	58	F	2.20	10	1.07	−30.2	4.58	+1.8	N/A	N/A	Ascites, oral intolerance	1,3c,10	34	Y	3
19^f^	62	M	1.94	7	1.50	−60.2	4.42	−0.5	N/A	N/A	AMS	2,3a,7,10	26	Y	18
20	54	M	1.35	3	2.54	−61.3	5.56	+2.4	4.75	5.0	Large volume paracentesis	2,3c,5,7,8,10	24	Y	36

Abbreviations: AMS: altered mental status; CKD: chronic kidney disease; EtOH: ethanol; GI: gastrointestinal; HRS: hepatorenal syndrome; IVCmax: maximum inferior vena cava diameter; MELD: Model for End-Stage Liver Disease; N/A: not applicable since patient received intermittent or continuous hemodialysis; SBP: subacute bacterial peritonitis; SCr: serum creatinine concentration.

Secondary disorders: 1= AKI, 2= AKI/CKD, Cirrhosis due to 3a = EtOH, 3b = Hepatitis B and/or C, 3c = Cryptogenic, 4= Hypertension, 5= Diabetes mellitus, 6= Chronic heart failure, 7 = Acute decompensated liver disease, 8= Infection, 9= Acute GI bleeding, 10= Oliguria or not quantitated, 11= Improved urine output with time, 12= Urine protein to creatinine ratio >0.5 g/g (1.85 g/g for pt#11, and 0.74 g/g for pt#14). Non-oliguria, urinary dipstick protein of <100 mg/dL or urine protein to creatinine ratio <500 g/g were not indicated. Urine sodium <20 mEq/L was not indicated since this was expected with HRS-AKI and values lower than <20 mEq/L are not reported by our laboratory.

^a^Initial IVC ultrasound after standardized albumin administration and diuretic withdrawal.

^b^Baseline serum creatinine is the lowest value available within 3 months prior to consult (1).

^c^Initial peak serum creatinine within 3 days of initial IVC US and during the time of the AKI episode.

**Figure 2.**
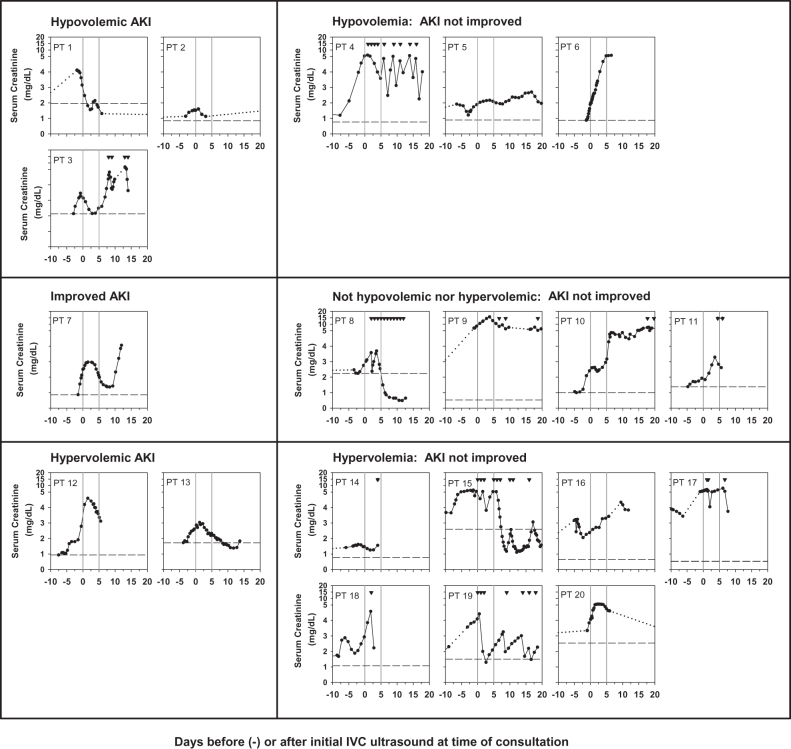
Changes in serum creatinine levels over time related to intravascular volume status categorized by IVC US as hypovolemia, not hypovolemia nor hypervolemia, or hypervolemia. Standardized albumin administration had been given with discontinuation of diuretics prior to initial IVC US assessment in all 20 patients. The phrase ‘AKI not improved’ means failure of serum creatinine to improve with volume management after the initial IVC US or requirement for HD therapy and thus HRS-AKI could not be excluded as a diagnosis. Patient numbers correspond to those in Table 2. Hypovolemia: IVC CI ≥50% or IVCmax ≤0.7 cm; not hypovolemic nor hypervolemic: IVC CI ≥20–<50% and IVCmax >0.7 cm; Hypervolemia: IVC CI <20% and IVCmax >0.7 cm. Solid lines connect serum creatinine values during hospital admissions, including the time when the initial IVC US was performed. Dotted lines connect serum creatinine values spanning an interval in which values were not available. Solid downward triangles at the top of the graphs indicate time(s) of intermittent hemodialysis or continuous venovenous hemodiafiltration. The *Y*-axis indicates the linear serum creatinine values from 0 to 5 mg/dL and from 5 to 20 mg/dL. The two vertical lines indicate the time of the initial IVC US and day five of subsequent follow-up. The dashed horizontal line in each panel indicates the baseline serum creatinine within 3 months prior to the consultation. Abbreviations: AKI: acute kidney injury; IVC US: inferior vena cava ultrasound; PT: patient.

^d^ Unable to exclude HRS-AKI based on the change in serum creatinine concentration within 4–5 days from the time of initial IVC US (See Figure 2).

^e^Not receiving hemodialysis therapy.

^f^Transferred for evaluation for liver transplant.

^g^Total venous collapse seen with respiration.

**Table 3.** Intervention and subsequent treatment within 5 days after initial IVC US.

Category of volemia	Pre-IVC US	Outcome within 4–5 days of follow-up	Treatment during 4–5 days post-IVC US		
	Albumin, no diuretics		Midodrine^a^	Octreotide^a^	Volume management
Hypovolemic (*N* = 6)	6 of 6	3 of 6 decreased SCr ≥20%	1 of 3	1 of 3	Albumin 75 g/day for 1–2 days for 3 of 3
		2 of 6 did not decrease SCr ≥20% 1 of 6 required HD	1 of 3	1 of 3	Albumin 75 g/day for 3 of 3 Isotonic saline 500 mL for 1 of 3
Not Hypovolemic nor Hypervolemic (*N* = 5)	5 of 5	1 of 5 decreased SCr ≥20%	1 of 1	0 of 1	Bumetanide 2 mg q12h IV and Metolazone 5 mg qd po for SOB
		2 of 5 did not decrease SCr ≥20% 2 of 5 required HD	3 of 4	4 of 4	Ongoing volume losses replaced as albumin, isotonic saline and/or blood products for 4 of 5 (12, 13)
Hypervolemic (*N* = 9)	9 of 9	2 of 9 decreased SCr ≥20%	1 of 2	1 of 2	Furosemide 80 mg q12h IV and metolazone 5 mg qd po for 1 of 2 Bumetanide 2 mg q12h IV and metolazone 5 mg qd po for 1 of 2 Paracentesis 0 of 2
		2 of 9 did not decrease SCr ≥20% 5 of 9 required HD/UF	4 of 7	4 of 7	Furosemide 80 mg q12h IV and metolazone 5 mg po qd for 3 of 7 Bumetanide 2 mg q12h IV and metolazone 5 mg po qd for 1 of 7 Paracentesis 0 of 7

Abbreviations: HD: hemodialysis; IVC US: inferior vena cava ultrasound; SCr: serum creatinine; SOB: shortness of breath; UF: ultrafiltration.

^a^Dose of Midodrine (5–20 mg tid orally) and Octreotide (50–100 mcg q8h subcutaeously).

None received intravenous norepinephrine during the 3 days of follow-up.

The changes in serum creatinine values before and after the renal consultation are shown in Figure 2. Changes in serum creatinine values were assessed for the first 4–5 days after the initial IVC US. Of the six patients with IVC CI ≥50% or IVCmax ≤0.7 cm, consistent with intravascular hypovolemia, who received additional volume administration, 3 had serum creatinine concentrations decreased by ≥20% without dialysis therapy and were categorized as hypovolemic AKI. The serum creatinine values tended to decrease in patient #1 and patient #3 before the IVC US was performed. Two of the other 3 hypovolemic patients failed to improve serum creatinine levels with volume administration and one of them required intermittent hemodialysis therapy within the 4–5 days; HRS-AKI could not be excluded for these three patients. Of the five patients with IVC CI ≥20 to <50% and IVCmax > 0.7 cm, inconsistent with intravascular hypovolemia or hypervolemia, patient #7 with shortness of breath who received diuretics had improvement in serum creatinine levels ≥20%, two patients required hemodialysis therapy and two patients’ serum creatinine values failed to decrease ≥20% by 4–5 days of follow-up. Thus for four of these five patients HRS-AKI could not be excluded. Of the nine patients with IVC CI <20% and IVCmax >0.7 cm, consistent with intravascular hypervolemia, who had volume restricted and received intravenous diuretics and/or ultrafiltration as tolerated, two had serum creatinine values persistently decreased ≥20% without dialysis therapy within the 4–5 days of follow-up and were categorized as hypervolemic AKI, and seven patients had persistently elevated serum creatinine values or required hemodialysis therapy during the first 4–5 days of follow-up, and HRS-AKI could not be excluded. Thus 6 of the 20 patients (30%) were diagnosed as AKI with intravascular volume depletion or overload and improved AKI within 4–5 days of follow-up with further volume management and for 14 of 20 patients (70%), a diagnosis of HRS-AKI could not be excluded (Figures 1 and 2, Table 2).

Eight patients received acute intermittent or continuous hemodialysis therapy within 4–5 days of follow-up for intractable volume overload, hyperkalemia, and/or uremia (Figure 2 and Table 2). Only 4 of the 20 patients were subsequently referred for possible liver transplantation (Table 2). Table 3 shows the therapeutic interventions within the 4–5 days of follow-up. None of the patients received norepinephrine infusion or large volume paracentesis during the 4–5 days of follow-up. After 5 days of initial follow-up, serum creatinine values improved in one of the hypervolemic group who also did not receive norepinephrine or terlipressin therapy.

Three of the 52 patients with potential HRS-AKI were excluded at the time of the renal consult and initial IVC US due to bladder pressures >20 mmHg which were secondary to hemoperitoneum in two patients and hemorrhagic pancreatitis in one, which may have caused or contributed to the AKI. IVCmax values for these three patients were 3.28 cm, 2.28 cm and 1.82 cm, respectively, and IVC CI values were 1.5%, 0% and 0% respectively, at the time of bladder pressure measurement.

## Discussion

To our knowledge, this is the second study reporting the frequency of IVC US-determined hypovolemia or hypervolemia in patients with cirrhosis and ascites who were assumed to have HRS-AKI by current criteria after failing to respond to standardized volume administration and diuretic withdrawal [1], and their subsequent response to additional IVC US-guided volume management.

Patients meeting current HRS-AKI criteria [1] are presumed to have intravascular volume repletion after standardized volume administration and diuretic withdrawal for at least 48 h. However, the presence of clinically undetected intravascular hypovolemia or hypervolemia may contribute to AKI in patients with cirrhosis and should be corrected in order to identify those with AKI that may respond to further volume management. After standardized volume administration followed by IVC US evaluation, we found that 30% of our 20 patients had an IVC CI ≥50% or IVCmax ≤0.7 cm, consistent with intravascular hypovolemia, and 45% had an IVC CI <20% and IVCmax > 0.7 cm, consistent with intravascular hypervolemia, with only 25% being considered as having appropriate volume repletion. In the study of 53 clinically diagnosed HRS-AKI patients reported by Velez et al. [9], using different IVC US criteria (Table 4), 28% were considered to have hypovolemia, 21% hypervolemia, 36% euvolemia, and 15% were defined as having IAH after volume repletion with the standardized protocol. If the intravascular volume status of our patients had been defined using the criteria used by Velez et al. [9], 12 of 20 patients (60%) would have been reclassified, as shown in Table 4. Subsequent volume management of each patient in both studies was based on the respective IVC US criteria used to classify intravascular volume and IAH at the time of the patient consult. Our finding of 45% hypervolemia, by our IVC US criteria, after standardized albumin administration is consistent with the report by Shasthry et al. [4], that 30% of 99 patients with cirrhosis who received an albumin infusion of 1 g per kg over 4 h developed clinical, echocardiographic and/or hemodynamic changes indicating hypervolemia.

**Table 4.** Comparison of presumed intravascular volume status, IVC ultrasound criteria, response to volume management, and frequency of possible HRS-AKI in the current study compared to the study by Velez et al. [9].

Intravascular volume categories	Current study			Study by Velez et al. [9]			Reclassification of our 20 patients using IVC criteria of Velez et al. [9] with outcomes
	IVC US criteria^a^	*n* (%)	Outcomes	IVC US criteria	*n* (%)	Outcomes	
		20	6 of 20 (30%) AKI improved		53	12 of 53 (23%) AKI improved	
Hypovolemia	IVC CI ≥50% or IVCmax ≤0.7 cm	6 (30%)	3 of 6 (50%) AKI improved	IVCmax <1.3 cm IVC CI >40%	15 (28%)	6 of 15 (40%) AKI improved	5 Classified as hypovolemic − 2 Improved with volume repletion 1 Reclassified as euvolemic – improved with volume repletion
Not Hypovolemia nor Hypervolemia	IVC CI ≥20% to <50% and IVCmax >0.7 cm	5 (25%)	1 of 5 (20%) AKI improved	IVCmax 1.3–2.0 cm	19 (36%)	0 of 19 (0%) AKI improved	2 Classified as euvolemic − 0 improved 2 Reclassified as hypervolemic − 1 improved 1 Reclassified as intra-abdominal hypertension^b^ – Did not improve
Hypervolemia	IVC CI <20% and IVCmax >0.7 cm	9 (45%)	2 of 9 (33%) AKI improved	IVCmax >2.0 cm IVC CI <40%	11 (21%)	3 of 11 (27%) AKI improved	3 Classified as hypervolemic − 2 Improved with volume removal 6 Reclassified as euvolemic – 0 Improved with volume removal
Intra-abdominal hypertension		3 with bladder pressure >12 mm Hg (excluded)	0 of 3 (0%) AKI improved	IVCmax <1.3cm IVC CI <40%	8 (15%)	3 of 8 (38%) AKI improved	2 Reclassified as hypervolemic 1 Reclassified as euvolemic – none improved

Abbreviations: CI: collapsibility index; HRS-AKI: hepatorenal syndrome with acute kidney injury; IVC: inferior vena cava; n: number; IVCmax: maximum IVC diameter.

^a^Log-likelihood ratio (LLR) *p* = 0.52 comparing total number of patients that improved or not in our study, exclusive of intra-abdominal hypertension, compared to all patients of Velez et al. (9), and *p* = 0.38 comparing those that improved or not in our study to those that did or did not improve in the study by Velez et al. (9) after excluding those patients meeting intra-abdominal hypertension criteria from both studies.

^b^IVCmax may be underestimated due to the cylinder tangent effect with a markedly distended abdomen and IVC collapsibility may be reduced due to low tidal volumes and/or with abdominal distention due to large volume ascites.

Improvement in serum creatinine values implies patient had volume responsive AKI; failure to improve implies unable to exclude HRS-AKI.

Standardized volume administration in patients assumed to have HRS-AKI by clinical criteria may frequently result in IVC US findings consistent with hypovolemia or hypervolemia, rather than the presumed appropriate intravascular volume repletion. These findings support the role of IVC US evaluation of intravascular volume, following the standardized protocol for volume administration in cirrhotic patients assumed to have HRS-AKI, to minimize persistent hypovolemia or occurrence of hypervolemia as a factor contributing to ongoing AKI.

Subsequent volume management, taking into account IVC US findings, may be associated with improvement in AKI in patients assumed to have HRS-AKI. In our study, 30% of patients assumed to have possible HRS-AKI had improvement of serum creatinine levels after additional volume management during follow-up for 4–5 days compared to 23% in the study by Velez et al. [9] (Table 4). Serum creatinine values tended to decrease prior to the IVC US in two of the three hypovolemic patients suggesting initial response to pre-IVC US volume administration or other factors. By the fifth day, serum creatinine values improved with additional volume management in three of six intravascularly hypovolemic patients, one patient classified as not hypovolemic nor hypervolemic with shortness of breath who was treated with diuretics, and two of nine intravascularly hypervolemic patients, without norepinephrine or terlipressin therapy, and were considered to have a diagnosis other than HRS-AKI [1,2]. In the study by Velez et al. [9], serum creatinine values improved 2 to 3 days after the therapeutic intervention in 6 of 15 patients designated as hypovolemic, in 3 of 11 hypervolemic and in 3 of 8 with IAH, consistent with a diagnosis of AKI other than HRS-AKI. The recovery of serum creatinine levels in patients in both studies initially assumed to have HRS-AKI could have been due to the volume management after the consult, recovery from acute tubular necrosis (ATN), or to other factors. In a randomized controlled trial of albumin plus terlipressin or placebo for the treatment of HRS-AKI [14], AKI was reversible without renal replacement therapy after 14 and 30 days in 17–18% of 101 patients receiving placebo, due to unstated reasons, compared to 32% of 199 patients treated with terlipressin. Similar improvement in renal function with placebo (15%) versus terlipressin (27%) was found in the pooled analysis of the REVERSE and OT-0401 trials [15] providing clear evidence of misclassification of HRS-AKI based on clinical criteria. None of our patients or those reported by Velez et al. [9] received terlipressin or norepinephrine therapy, or liver transplantation prior to improvement in AKI.

Three of our initial 52 patients with potential HRS-AKI were excluded due to a diagnosis of IAH with bladder pressures >20 mmHg, which may have exacerbated AKI. In the study of Velez et al. [9], 8 of 53 patients had IAH, defined as IVCmax <1.3 cm and IVC CI <40%, without confirmatory bladder pressures recorded. Two of our included patients met the US criteria for IAH used by Velez et al. [9], and neither had clinical evidence of IAH however bladder pressures had not been measured. All three of our patients who had documented IAH by bladder pressures and who were not included as possible HRS-AKI had an IVCmax >1.8 cm and IVC CI <2% without obvious focal narrowing on IVC US, suggesting that IAH may not decrease IVCmax below 1.3 cm when patients have intravascular volume overload. Bauman et al. [16] showed that in patients undergoing laparoscopic procedures, IVCmax decreased significantly with increasing intra-abdominal pressure but there was no correlation of intra-abdominal pressure with IVC CI.

Our study supports the findings of Velez et al. [9] that intravascular volume assessment by IVC US in patients meeting established criteria for HRS-AKI [1,2] may make the diagnosis of HRS-AKI more precise and guide volume management to correct overt hypovolemia or hypervolemia, which in turn may improve AKI in patients with cirrhosis and ascites. A search for potentially reversible causes of AKI in patients with cirrhosis using IVC US to assess intravascular volume followed by further volume management is important since the treatment and prognosis of assumed HRS-AKI differs from that of other causes of AKI in patients with cirrhosis [1,17].

The limitations of our study are numerous. It is a retrospective study. The number of patients was small with only 20 patients identified over 8 years resulting in the potential for selection bias. IVC US was primarily performed in the sickest cirrhotic patients with AKI who frequently had multi-organ dysfunction and other possible causes of AKI resulting in only a very small number of patients who met HRS-AKI criteria, which is a diagnosis of exclusion. IVC US findings may be user dependent, however studies were performed or directly supervised by one experienced physician. Differentiation of HRS-AKI from ATN-AKI was not possible. Bladder pressures were only measured in patients with a bladder catheter who were suspected to have IAH. An additional limitation was the difficulty in visualizing the IVC due to abdominal distention and/or pain, and poor liver window due to cirrhosis or acute hepatitis in an undetermined number of patients.

## Conclusion

Assessment by IVC US revealed 15 of 20 (75%) patients clinically diagnosed as possible HRS-AKI were intravascularly hypovolemic or hypervolemic after standardized albumin administration and diuretic withdrawal and six of these had improvement of AKI with further volume and medical management. Therefore, 6 of 20 (30%) patients meeting current criteria for HRS-AKI were shown to have AKI which may have been responsive to additional ultrasound guided volume management. IVC US to evaluate intravascular hypovolemia or hypervolemia after standardized volume administration and diuretic withdrawal may indicate when further volume management may be clinically relevant to accurately diagnose and manage patients with cirrhosis and AKI, and may be useful to assess response to interventions and outcomes of clinically diagnosed HRS-AKI in future studies. Perhaps performing an IVC US to assess intravascular volume before, during and/or after administering the standardized volume therapy with discontinuation of diuretic therapy, would be beneficial to avoid prolonging hypovolemia or inducing hypervolemia, and may improve clinical outcomes. Decreasing the risk or severity of AKI may potentially reduce the morbidity and mortality in patients with cirrhosis [3].
